# Towards a new way of interacting? Pondering the role of an interpersonal ethos

**DOI:** 10.1186/s40878-018-0081-7

**Published:** 2018-05-17

**Authors:** François Levrau

**Affiliations:** University of Antwerp, Prinsstraat 13, 2000 Antwerp, Belgium

**Keywords:** Interpersonal ethos, Egalitarian ethos, Multiculturalism, Interculturalism, Integration, Caring interactions

## Abstract

Both multiculturalism and interculturalism strive for the creation of a well-ordered society in which the integration of all citizens is realized. This shared ambition, however, can only be realized if the quality of the social relationships is deeply considered. By proposing the concept of 'interpersonal ethos' it is explained why and how the way ordinary people socially interact across ethnocultural and religious diversity shapes the stability, success and fairness of a super diverse society.

## Introduction

As one of the most controversial ideas in contemporary liberal politics and philosophy, multiculturalism has given rise to a vast array of literature that covers a wide range of issues, perspectives and discussions. Most scholars discuss, add or reject certain ideas within the multicultural framework or they engage with some canonical multiculturalists or texts in order to point out the flaws of the specific theory they are scrutinizing. Scholars like Ricard Zapata-Barrero, however, no longer defend their ideas under the banner of multiculturalism, implying that their ideas are so fundamentally different, that they cannot be subsumed under the multicultural flag and thus need another/new signifier, or even be the starting point of another/new discipline. While Zapata-Barrero (2017) does not dismiss all elements of the multicultural framework, he maintains that interculturalism deserves all credentials for being a new and different paradigm that is much more suitable to deal with the challenges of a super-diverse society. Modood (2017), on the contrary, contends that there is no need for interculturalists to push the issue too hard, as they might saw off the very branch upon which they themselves are sitting, a diagnosis that is shared by Kymlicka (2016). Elsewhere, we have argued why both approaches may be different, but more importantly, why they need to be considered as complementary strategies to create just and equal societies (Levrau & Loobuyck, 2013; see also Loobuyck, 2016). We will not repeat these arguments, but move the spotlight to one particular issue that might not always have been given full consideration in the ‘multiculturalism-interculturalism debates’.

Our main point is that multiculturalism and interculturalism can only be successful in their shared ambition (i.e. the creation of a well-ordered and stable society in which the integration of all citizens is realized on an equal basis) if these citizens act on the basis of an interpersonal ethos that guides their many daily choices, interactions and behaviours. We contend that the alchemy wrought by a fair basic structure with laws, rules and institutions that protect individuals and minority groups against the power of particular individuals, groups and the state as such, needs to go further, insofar as people should also act justly in their individual lives. This would have been self-evident for the ancient philosophers, as political principles were simply ethical principles writ large. The aim of a just society – take Plato’s *Republic* as a good example – was to make people virtuous. For modern liberal philosophers, however, political philosophy is not congruent with ethical philosophy, as its main goal lies in the creation of appropriate institutions within whose rules people can/may pursue their diverse and often opposing ends.

We start with a brief presentation of the Cohen-Rawls debate, as Gerald Allen Cohen made the particular claim that citizens must be motivated by an ‘egalitarian ethos’ so that they act in their daily economic activities and choices in accordance with the institutional principles that ensure that society is characterized by equality. As the Cohenian ethos operates within the distributive sphere, we argue that the social relations in a diverse society also need to be informed by a specific ethos. We contend that an egalitarian-institutional concept of (multicultural/intercultural) justice needs a corresponding psychology where empathy and concern are pivotal. We will consider the role of the ethos and examine how it relates to the multicultural approach of Modood and the intercultural frame of Zapata-Barrero. We argue that the latter has more potential for discounting the particular role of the interpersonal ethos.

## Rawls vs. Cohen

In Rawls’s *original position*, people are asked how society’s basic structure is best designed to be qualified as just. Since people tend to favor themselves, they are placed behind the ‘veil of ignorance’. According to Rawls (1971), people will come to the conclusion that everyone must have equal and maximum freedom that is compatible with equal freedom for others. Everyone must have an equal opportunity to shape his life according to his/her own conception of what ‘the good life’ presupposes. Socioeconomic inequalities may exist, provided that this inequality is in everyone’s favor and does not harm society as a whole (the famous ‘difference principle’). The stability of Rawls’s society depends largely on the extent to which the aforementioned justice principles are accepted. According to Rawls this is indeed the case, since people have a ‘sense of justice’, an ability to understand and maintain the principles of justice in service of fair social cooperation.

In contrast to Rawls, Cohen has argued that the justice of a society not only depends on the basic structure, but also on the many (daily) actions and choices of citizens. *A society that is just within the terms of the difference principles (…), requires not only just coercive rules, but also an ethos of justice that informs individual choices* (Cohen, 2008, p. 16). Cohen does not agree with Rawls’s ‘difference principle’, which allows the ‘talented’ to earn more provided that they promote the position of the ‘weakest’. According to Cohen, Rawls’s interpretation of the difference principle primarily expresses the will of the most privileged. After all, they can blackmail the weak and thus the society as a whole. If the ‘talented’ were truly devoted to the equality that the difference principle intends, they would apply it in a stricter manner, only asking for extra financial incentives if proven strictly necessary. Cohen proposes the ‘interpersonal test’ for talented citizens to justify their extra financial rewards. *“An argument fails the interpersonal test, and is therefore inconsistent with community, if relevant agents [such as talented rich person] could not justify the behavior the argument ascribes to them”* (Cohen, 2008, p. 44). According to Cohen, the talented cannot justify why they need additional incentives if they can achieve the same productivity without incentives. The ‘rich/talented’ who requests incentives must therefore be considered as an outsider of the community or as an insider who behaves unethically. Indeed, he is not focused on the creation or preservation of a moral climate that makes society ‘egalitarian’ and, therefore, according to Cohen, ‘just’. A just society must cultivate an egalitarian-economic ethos that determines personal behaviour.

Cohen’s ‘egalitarian ethos’ was criticized by various authors, the main criticism being that the extension of principles of justice to the individual/social life implies an impediment to individual freedom. Cohen, however, does not suggest that talented people should give up their freedom. The point seems to be that every member of society bears a moral duty to be aware that his actions and choices can be justified to a greater or lesser extent and thus be directed by ‘equality’ as a pole star. Carens (2015) suggests the Cohenian ethos could be seen as a social mechanism. People should not be fined if they act differently – penalization belongs rather to laws and regulations – but the ethos produces socially compelling effects: people are effectively ‘punished’ by public morality, which criticizes and rejects certain behaviour.

## Multiculturalizing the basic structure vs. the interpersonal ethos

As was the case with the discussions related to the ‘politics of redistribution’, the vast majority of attention in the multiculturalism debate has gone to the basic structure of society. Most multicultural scholars have argued why governments are not culturally neutral, even though their justifications for policy and law might live up to a neutrality of justification. They have also brought the whole discussion to another level by asserting that Rawls, Dworkin and in fact a whole generation of liberal theoretists were blind to this cultural bias. As a neutral government should treat all its citizens with equal respect and concern, multicultural measures or rights for ethno-cultural groups should be instituted to ensure equal opportunities and to enhance the fair integration of ethno-cultural minorities who want to live according to their own traditions and religions. While it would be bad faith to believe Modood, as one of the ‘multicultural champions’, has neglected the specific role of the citizen or that multiculturalism has nothing to say about citizen-to-citizen relations (see e.g. Modood, 2006, 2010), we take it as a fact that it was not given equal attention to the justification of adjustment of the basic structure.

Zapata-Barrero (2016, 2017) does not necessarily reject the multicultural rights-based and duties-based policy approaches, but he emphasizes more the need for actual contact-based policies, aimed at fostering communication and relationships among people with different backgrounds. As his interculturalism approach is said to be a technique of bridging differences, bonding and promoting social capital in public spheres of people’s everyday lives, it acknowledges the role of daily interactions. At this point, Zapata-Barrero (2017) explicitly refers to the fact that individuals need to be seen as *‘holders of competences that need to be promoted’* (p. 11). He also refers to the need for an *‘intercultural mindset’* (p. 7). While he has not explained in much detail the status of this mindset nor what it exactly entails, we believe it might be characterized or determined by an ‘interpersonal ethos’.

Pondering the role and importance of the ethos seems crucial as people’s everyday conduct, choices and interactions in civil society also contribute to the fairness, success and sustainability of a society. After all, why should we attach disproportionate importance to the compelling influence of the basic structure? The impact of the basic structure on people’s lives is clear, but the informal structure has an equally significant impact on people’s prospects. Rawls, for example, gave the basic structure primacy because its effects are so fundamental, but the interactions, decisions, choices, and behaviours of people within that society often have an equally profound effect. If the ambition of multicultural and intercultural theories is to outline the normative and political frame of a just and fair society, we believe the role of the ethos needs to be taken seriously, as it might be essential for the realization of such a society. People, for example, can block racist parties or racist laws and rules through their ‘sense of justice’, but that does not mean that they are thereby preventing ‘latent’ racist behaviour manifesting in their daily lives. Would anyone call a society non-racist if there are many antiracist laws, but in practice, people exhibit all sorts of (latent) racist behaviour? The ‘multiculturalization’ of the basic structure by no means implies that citizens will behave in their daily interactions and choices in accordance with the principles of justice that support this institutional multiculturalization; namely, treating each person with equal respect and concern. The stability, and I would add, the fairness of a society also depends on how people behave, and this behaviour presumes much more than the occasional vote for non-racist parties and support for anti-racist or multicultural laws, rules and institutions. As the ethos is a crucial element for a just and well-ordered society, a multicultural or intercultural theory of justice that does not consider its role and impact cannot be complete. Multiculturalists, however, have typically treated the ethos in a rather ‘stepmotherish way’ (good exceptions, however, are Laegaard, 2011 and Levey, 2010). Take Kymlicka (2016, pp. 172-173) who argues: *“It may well be true that we would all be better off, individually and collectively, if we were more able and willing to engage in intercultural dialogue, but it’s difficult to see why this is an issue of justice or collective obligation. Multiculturalism by contrast highlights the problem in the state-sponsored privileging of nationhood, and in the exclusions this had entailed, which makes clear why it is a matter of justice and collective responsibility.”* This quote is illustrative for how multiculturalists underscore the basic structure and somewhat neglect the importance of day-to-day interactions. Interculturalists like Zapata-Barrero (2016), on the other hand, have proposed a theoretical frame in which the (conditions for) daily ‘multicultural relations’ are crucial. Incorporating the interpersonal ethos then seems a self-evident aspect of this theory.

We believe the ‘multiculturalization of the basic structure’ can be justified by the original position and the veil of ignorance: the precise form of this multiculturalization can often be found through dialogue, as Modood (2017) has rightly argued, and can eventually lead to the kind of interculturalization or mainstreaming that Zapata-Barrero (2017) proposes. It is, however, also the interpersonal test of Cohen that justifies the interpersonal ethos. As such, interculturalism can be defended via Cohen’s interpersonal test where concrete people meet and wonder whether, for example, they have a good reason not to sit beside a veiled lady, not to go to the Turkish neighborhood shop, not to send children to a school with many migrant children, etc. The dialogue as proposed by multiculturalists like Taylor, Parekh and Modood is first and foremost an institutional and policy-driven dialogue – where negotiations deliver fair laws and practices. The intercultural dialogue as proposed by Zapata-Barrero, on the other hand, governs more the informal sphere where ordinary people engage in face-to-face communication and make choices in real-life situations.

## Putting flesh to the bones of the interpersonal ethos

So far, we have been rather vague about the ethos. Let us thus consider some examples in order to flesh it out a bit.

Imagine a man getting on the train. There are two seats available: he can sit either next to a woman wearing a headscarf or next to a woman without a headscarf. Our man prefers the latter, because he believes that people with headscarves are inferior and cannot be trusted. While the behaviour of the man is morally disrespectful insofar as he fails to treat the woman as an equal, it is not entirely clear if his behaviour is also unjust. After all, he is free to choose where to sit. Moreover, as the woman will probably never know that the man has purposefully avoided her, she is in no way disadvantaged. We will skip the tricky question of whether or not justice depends on the awareness, as it bears some resemblance to the ancient question of whether or not a tree falling in a forest makes a sound if there is no one to hear it, and will instead look at the situation where the woman knows that the man has deliberately avoided her. In that case, she will feel humiliated and will suffer the resulting drop in her self-respect. At this point, one might invoke that the single act of our man is unproblematic, as long as there are plenty of others that would not mind sitting next to a veiled woman. However, if plenty of people would avoid her, she will feel excluded and will eventually withdraw from society. This feeling of ‘not being a part of society’, however, is precisely what multicultural and intercultural authors want to tackle with the ‘multiculturalization’ or ‘mainstreaming’ of the basic structure. A society where citizens do not treat each other with equal respect will not be experienced as a just society. Even if each individual behaved only slightly injustly, e.g. avoiding a woman with a headscarf, the end result might still be an unjust society. Kernohan (1998), for example, has pointed out that ‘cultural oppression’ acts as cumulatively and nebulously as air pollution. The harm caused to our fundamental interest in self-respect is analogous to environmental pollution, and though often difficult to detect, it is just as real. Once a certain pattern has been established, even individual acts can have harmful effects, as they are seen as the result of a pattern. Literature on ‘stereotype threat’, for example, has illustrated that students who become aware that ‘their’ culture is seen as inferior have high risks of conforming to the stereotypes and thus (unconsciously) deteriorate their own life prospects. This means that a state that is committed to the moral equality of persons must accept a role in reforming the cultural environment by stimulating an interpersonal ethos as a social mechanism to guide people’s daily behaviour. If not, society, at least in its consequences, will not be fair.

Another example is the Golem effect, whereby the low expectations of a teacher negatively influence the effective performance of students. If a teacher is convinced that a student is a ‘low performer’ the effect might be realized in a self-fulfilling way. The teacher’s bias may not be obvious even to himself, but the lack of equal respect can result in certain students being given fewer opportunities for educational and consequently professional success. Although the teacher may not always be aware of this – here the question might again be posed whether justice depends on awareness, but now on the side of the ‘perpetrator’ – it should be clear that what he is doing is not only morally incorrect, but also unjust, as the consequences for the students are severe.

Yet another example of what an interpersonal ethos entails is related to the right to ‘free speech’. As there is no such thing in a liberal society as a right not to be offended, any effort at censorship that goes beyond the well-known limitations of hate speech and the like — whether state enforced or self-imposed — would be a serious setback for freedom of expression. Therefore, people have to accept that they cannot be equally esteemed and that others may criticize their deepest convictions and ways of life. The interpersonal ethos, however, will take into consideration the vulnerability of people beyond the physical integrity. People not only have to take into account the physical integrity, but also the psychological integrity of Nietzsche’s ‘animal with red cheeks’. Indeed, just as Cohen criticized Rawls for a lax interpretation of the difference principle – it is advantageous for the strongest party and seems to be rather a principle that deals with the moral brutality of people – one might think that freedom of expression is all too often invoked to justify the moral brutality of the ‘strongest’. This does not contradict that the freedom of speech is meant for the ‘weakest’ – in the sense that they treasure unpopular ideas and thus should be guaranteed the right to defend unconventional convictions. The point, however, is that under the motto *“Sticks and stones may break my bones, but words can never hurt me”*, one can often manifest Shakespeare’s dictum *“I’ll speak daggers to her.”* The interpersonal ethos will dissuade people from formulating critique in a way that humiliates. Obviously, people do not have to be equally esteemed, but the way in which the lack of esteem is expressed can go hand in hand with a distortion of the moral equality of people. The interpersonal ethos thus does not restrict freedom of expression but rather attempts to bring ‘freedom’ into relation with ‘equality’ and ‘solidarity’. It is one thing to lawfully address hate speech (an element of the just basic structure), but the focus on hate speech purloins the importance of (elementary) respect, which expects a sensitivity and social-emotional intelligence asking people to take into account the human vulnerability (an element of the interpersonal ethos).

## The utopia of caring interactions?

The ethos has a deep account of sensibility for specific characteristics of situations that can be captured through concepts of respect, concern, integrity, dignity, self-esteem, honor, politeness, vulnerability, empathy, civic friendship, care, trust, solidarity, appreciation, openness, gratitude, decency. Attention towards these concepts might eventually lead to what Margalit (1996) has coined the ‘descent’ and the ‘civilized’ society. People driven by an interpersonal ethos illustrate a sensible psychology and hold ‘concern’ as an element that trumps other behaviour that might be lawfully allowed. Given the mutual dependency and hence the fundamental role of ‘recognition’ to the psychological makeup of men, we believe it is of high importance to favor a certain conception of the relations among modern citizens (Brudney, 2013). The basic premise should be that people think of themselves as autonomous beings capable to be concerned with others and, simultaneously, capable of acting from such concern. They need to see the exercise of this ‘concern capacity’ as a part of their good. Moreover, knowing that people, for example, not only pay their taxes out of compliance with some rules, but also because they really care for each other, coheres with a more satisfying form of social life. Experiencing that people not only vote against racist parties and policies, but also treat each other as moral equals, not only bolsters self-worth, but also increases mutual respect and tolerance.

Reflecting on everyday norms of social interactions, multicultural manners, or rather the conditions under which it would be rational for me to be concerned for the other and to believe that the other has also feelings of concern for me, seem to be more of an ‘intercultural’ than a ‘multicultural’ issue. However, it remains an important question whether this ethos is utopian and whether it can ever be able to function as a social mechanism that truly informs the attitudes and behaviours of people. Instantiating ‘the better angels of our nature’ is certainly not easy, given today’s rapidly changing societies where people (1) have different or even conflicting opinions on how collective life should be organized, as informed by their different socioeconomic and/or ethical backgrounds; (2) are in the grip of populist ‘politics of fear’ which drives people back in their own shells; and (3) are confronted with a dominant neoliberal discourse that emphasizes egoist reflexes and feeds the ‘Hobbes’ wolf’ rather than elevating the *‘homo socialis’*. One must, obviously, have sufficient reason to believe that others – who will for the most part remain strangers to us – are also motivated by the same type of ethos. Again, in an era where socioeconomic inequality is rising (Why would the poor trust the rich?), where all sorts of paranoiac and anxious feelings are being triggered (Why would we trust the poor and not believe they deserve their faith and are we really sure Muslims are not destroying Western civilization?), it will clearly not be an easy task to implement and promote an ‘ethos of concern’. However, if these types of questions are left unaddressed, social life may fall prey to growing disturbance and chaos.

As Kymlicka (2016) argues, multiculturalists and interculturalists must be aware that their conflicts do not provide grist for the mill of populists. Multiculturalists and interculturalists instead need to join forces in the creation of comprehensive policies that appeal to all citizens, as each citizen needs to be ‘integrated’ in the current and future super-diverse societies. This is obviously not the place to elaborate this element, but the success of these policies could benefit from the three classic modes of persuasion: ‘logos’, ‘pathos’ and ‘ethos’, where the mind, heart and soul of all citizens are touched. By ‘logos’ we mean a fuller acknowledgement of what is implied by ‘living in super-diverse and liberal-democratic societies’. People need, however, not only to be informed, also their social-emotional intelligence needs to be stimulated. ‘Pathos’ thus implies a proper and broad education of the sentiments. As such, a specific type of civic education that is non-coercive is of importance (Fives, 2013). ‘Ethos’ refers to the fact that dignitaries have to show the right example (‘Walk the talk’). The ‘interpersonal ethos’ obviously is not legally enforceable as the government would lose its liberal neutrality and hence would become paternalistic, but it would be meaningful if politicians demonstrate integrity in line with this ethos. After all, if one wants people to assume responsibility and to act upon the interpersonal ethos, they need to be inspired. A society in which the elites do not demonstrate exemplary behaviour will eventually generate the fading away of elementary respect and of ‘multicultural manners’ that are so crucial for the viability of a complex society.

## Conclusion

While ‘multiculturalism’ remains a container concept, we think it is fair to see it as a paradigm that is first and foremost about rights, recognition, and the adjustment of the basic structure so that it is equally open for ethno-cultural minority groups. As such, the multicultural citizenship approach is mainly focused on marginalization, segregation, cultural isolation from mainstream society and the misrecognition of ethno-cultural minority identities. While Modood obviously has not neglected the role of and need for interaction between ordinary citizens, it seems that Zapata-Barrero has brought this particular element much more to the fore. His intercultural paradigm therefore seems to be better equipped to fully account for the role and promotion of an ‘interpersonal ethos’. A deep elaboration of the status, role and character of the interpersonal ethos and how it can be promoted is likely to be more easily picked up by interculturalists, as they reflect on the preconditions of mutual respect behind daily interactions in such a way that contact and cohesion is more likely to be effective.
